# Genetic Characterization of Piroplasms in Donkeys and Horses from Nigeria

**DOI:** 10.3390/ani10020324

**Published:** 2020-02-18

**Authors:** Idoko Sunday Idoko, Sharon Tirosh-Levy, Monica Leszkowicz Mazuz, Babagana Mohammed Adam, Bello Sikiti Garba, Daniel Wesley Nafarnda, Amir Steinman

**Affiliations:** 1 Department of Veterinary Pathology, University of Abuja, Abuja 900001, Nigeria; idoko.sunday@uniabuja.edu.ng; 2 Koret School of Veterinary Medicine, The Hebrew University of Jerusalem, Rehovot 7610001, Israel; sharontirosh@gmail.com; 3 Division of Parasitology, Kimron Veterinary Institute, Bet Dagan 50250, Israel; monical@moag.gov.il; 4 African Institute of One Health Research and Diagnostics, Abuja 900001, Nigeria; babaganamb@hotmail.com; 5 United Nations Children Fund, Yola 640101, Nigeria; bsikiti@yahoo.com; 6 Department of Veterinary Public Health and Preventive Medicine, University of Abuja, Abuja 900001, Nigeria; wesley.nafarnda@uniabuja.edu.ng

**Keywords:** *Theileria equi*, *Babesia caballi*, donkeys, horses, Nigeria

## Abstract

**Simple Summary:**

*Theileria equi* and *Babesia caballi* are blood-parasites of horses and donkeys that are transmitted by ticks and may cause severe clinical illness. Many infected animals are carriers of parasites without showing signs of disease and, thus, pose a risk of transmission. Nigeria is a major passageway in animal import, export and transport within Africa. This movement of animals may play a key role in the spread of parasites. The aim of this study was to characterize these parasites that infect both horses and donkeys in Nigeria. Blood was collected from horses and draught-donkeys at two separately-owned farms in northern Nigeria. Infection with *T. equi* was detected in both donkeys and horses, with four of the five known genotypes present in Africa. Infection with a single genotype of *B. caballi* was detected in donkeys. Our results suggest that donkeys may be an important reservoir of these parasites. The high diversity of *T. equi* supports the hypothesis that animal transport through Nigeria may contribute to the spread of parasites to and from other countries in the region.

**Abstract:**

Equine piroplasmosis (EP) is a tick-borne disease of equids, caused by the two haemoprotozoal parasites: *Theileria equi* and *Babesia caballi*. Nigeria constitutes a major crossroads of animal transport in West Africa and may serve as a factor in EP dissemination in the region. The study aim was to characterize EP parasites in donkeys and horses in northern Nigeria using a molecular approach. Blood was collected from 57 donkeys and 47 horses. EP infection was detected and characterized by polymerase chain reaction (PCR). Twenty five donkeys (43.8%) were infected with *T. equi*, five (8.8%) with *B. caballi*, three (5.3%) with dual infections. Four horses (8.5%) were infected by *T. equi* and none by *B. caballi*. Four of the five known *T. equi 18S rRNA* genotypes (A, B, C and D) were identified. *Theileria equi ema-1* and *ema-2* genes were amplified in only 2 and 10 samples, respectively, showing no genetic variation. All *B. caballi* isolates were classified as *rap-1* genotype A1. Twenty-two (42.3%) of the donkeys were positive for anti-*T. equi* antibodies and 29 (55.8%) were positive for anti-*B. caballi* antibodies, using immunofluorescence antibody test (IFAT). The study results demonstrate high genetic variation within *T. equi* parasites, suggesting that donkeys may be reservoirs of EP parasites in West Africa.

## 1. Introduction

Equine piroplasmosis (EP) is a tick-borne disease of equids with worldwide distribution, mostly endemic in tropical and subtropical regions of the world [1,2,3]. The disease is caused by the intraerythrocytic protozoans, *Babesia caballi* and *Theileria equi*, and is of major negative economic significance [3,4]. Infection can be clinical or subclinical with the tick-transmitted haemoprotozoan parasites and can persist even after resolution of clinical signs [3,4]. The primary means of transmission is through the saliva of infected Ixodid ticks during a blood meal; other means of infection include transplacental and iatrogenic transmission through blood transfusion and/or contaminated needles [3,4,5]. The predominant vectors of EP are wide-range tick species of the genera *Amblyomma*, *Dermacentor*, *Haemaphysalis*, *Hyalomma* and *Rhipicephalus* [6]. Four of these five genera have been documented in Nigeria [7]. Equine piroplasmosis, which is endemic in Nigeria, has, until now, been documented mostly in horses [8,9,10,11,12,13].

The socioeconomic significance of donkeys in Nigeria is being threatened by rising rates of illicit slaughter for the skin trade and death by disease. Movement of donkeys across borders in most African countries, particularly those in sub-Saharan Africa, has been increasing in recent decades. The major hubs are Nigeria, Burkina Faso, Ghana, Mali and Senegal [14]. The transboundary movement of donkeys is a cause of veterinary and public health concern, considering the potential introduction of foreign pathogens and vectors. With a population of around two hundred million people, Nigeria is a destination for the massive influx of livestock including donkeys from neighboring countries. Donkeys are transported to different areas for use as draught-animals, as well as for the skin trade, and for meat.

The epidemiological data on EP in Nigeria were based solely on microscopic observation [8,9,10,12,13] and in a few cases, serology using competitive-inhibition enzyme-linked immunosorbent assay (cELISA) [11]. Microscopic diagnosis is not a sensitive method, especially in subclinical animals [15,16]. The commercial cELISA kit failed to detect *B. caballi* strains from Africa and the Middle East in horses that had been confirmed positive using molecular methods [17,18,19]. The reasons adduced for these failures were diversity in the rhoptry-associated protein-1 (*rap-1*) gene repertoire and considerable differences between these isolates and the Florida strain which was used to develop the monoclonal antibody (Mab) for the commercial cELISA kit [20].

Although EP is considered endemic in Nigeria, there is a lack of evidence on the prevalence of these parasites in naturally infected donkeys and horses in Nigeria, and no molecular studies have been published to characterize local parasite strains. The present study was designed to characterize the genetic diversity of *B. caballi* and *T. equi* parasites infecting horses and donkeys in northern Nigeria.

## 2. Materials and Methods

### 2.1. Study Population and Sample Collection

Blood samples were collected during 2019 from 57 donkeys and seven West African Barb horses from Zaria city (11.0622236, 7.706379), and from 40 Thoroughbred horses at a polo club (10.541005, 7.444234), in Kaduna state, Nigeria. At the time of sample collection, all animals appeared to be healthy and did not demonstrate any signs of disease. Blood was collected from the jugular vein of each animal into a sterile ethylenediamine tetraacetic acid (EDTA) vacutainer and a plain sterile tube without anticoagulant. Sera were separated from the clotted blood after 30 min of standing, transferred into 1.5 mL Eppendorf tubes and stored at −20 °C until analysis. The study was approved by the Ethical Committee, Faculty of Veterinary Medicine, University of Abuja, Nigeria. Blood samples were collected following owners’ informed consent.

### 2.2. Identification of Equine Piroplasmosis Parasites Using Polymerase Chain Reaction (PCR)

DNA was extracted from 100 µL of whole blood from each sample, using a commercial kit (DNeasy blood and tissue kit, QIAGEN, Hilden, Germany), according to the manufacturer’s instructions.

EP infection was detected using conventional PCR (C1000 Touch Termal Cycler, Bio-Rad, Haifa, Israel) directed at the *T. equi 18S rRNA* gene [21] and *B. caballi rap-1* gene [22] (Table 1), as previously described [19,23,24].

Positive samples for the *T. equi 18S rRNA* gene were further analyzed using PCR specifically targeting *T. equi ema-1* and *ema-2* genes (Table 1).

All positive PCR products were cleaned using Exonuclease I and Shrimp alkaline phosphatase (New England Biolabs Inc., Rehovot, Israel) and sent for sequencing (Macrogen Europe, Amsterdam, The Netherlands).

### 2.3. DNA Sequencing and Analysis

Sequences were evaluated using the Chromas software version 2.6 (Technelysium Pty Ltd., South Brisbane, Australia), and trimmed to leave good quality sequences. A few sequences of poorer quality were re-sequenced using the reverse primer, and a consensus sequence was constructed from the sequencing results of both primers using MEGA7 software (http://www.megasoftware.net, version 7.0.18). All good-quality sequences were submitted to the GenBank (accession numbers: MN093895, MN093923 for *T. equi 18S rRNA,* MN519202, MN519205 for *T. equi ema*-2 and MN105077, MN105081 for *B. caballi rap-1*). The sequences were 99–100% identical to previously published sequences of *T. equi 18S rRNA* gene or *B. caballi rap-1* available in GenBank.

### 2.4. Phylogenetic Analysis and Genotyping of T. equi Sequences

*Theileria equi 18S rRNA* sequences from all samples were aligned along with 22 *T. equi* sequences previously characterized, representing all five previously described genotypes, and other apicomplexan parasites available in GenBank.

Alignment was constructed via MEGA7 software, using the muscle function [26], and sequences were trimmed on both ends to receive a comparable sequence of 363 bp.

The phylogenetic tree was constructed using three different algorithms: Maximum Likelihood, Neighbor Joining and Maximum Parsimony, using Kimura 2-parameter +G+I model, selected based on best BIC (Bayesian Information Criterion) score, with 1000 bootstrap replicates to estimate reliability. Analysis was performed using MEGA7 software, and all models yielded similar results.

### 2.5. Detection of anti-T. equi and anti-B. caballi Antibodies Using Immunofluorescence Antibody Test (IFAT)

Serological screening for the presence of anti-*T. equi* and anti-*B. caballi* antibodies was performed using IFAT. The antigens were prepared in house from cultures and stored in −80 °C until use. Slides were dried at 37 °C for 30 min, fixed in acetone for 10 min and air dried before use. Sera were diluted to 1:80 and 1:160 with bovine serum albumin (BSA), with 1:80 considered as the cutoff value. A volume of 35 µL of serum was added to each antigen-containing well, incubated in a humid chamber at 37 °C for 30 min and later washed for 10 min in Phosphate buffer solution (PBS). A volume of 35 µL anti-horse antigen diluted 1:80 with BSA was applied to each well, and incubated at 37 °C for 30 min in a humid chamber. Slides were later washed for 10 min in PBS, dried, mounted with glycerol/PBS 1:1 and inspected under a fluorescent microscope. Positive and negative control samples were added to each run.

## 3. Results

### 3.1. Study Population

A total of 57 donkeys and 47 horses from Kaduna state, in Nigeria, were sampled. The donkeys were draught-animals used for carrying goods while the horses were used for pleasure riding and durbar (traditional ceremony). All 57 donkeys and seven horses from Zaria city were males. Horses from Kaduna were Polo horses, and included 15 (37.5%) males and 25 (62.5%) females (Table S1).

### 3.2. Equine Piroplasmosis Infection in Donkeys

Twenty-five (43.8%) donkeys had *T. equi* infection, five (8.8%) donkeys had *B. caballi* infection, three (5.3%) of these donkeys were co-infected with *B. caballi* and *T. equi* by PCR. Serological analysis of exposure was performed on 52 of the samples with available sera. Twenty-two (42.3%) of these samples were positive for anti-*T. equi* antibodies and 29 (55.8%) of these samples were positive for anti-*B. caballi* antibodies, using IFAT (Table S1).

**Figure 1:**
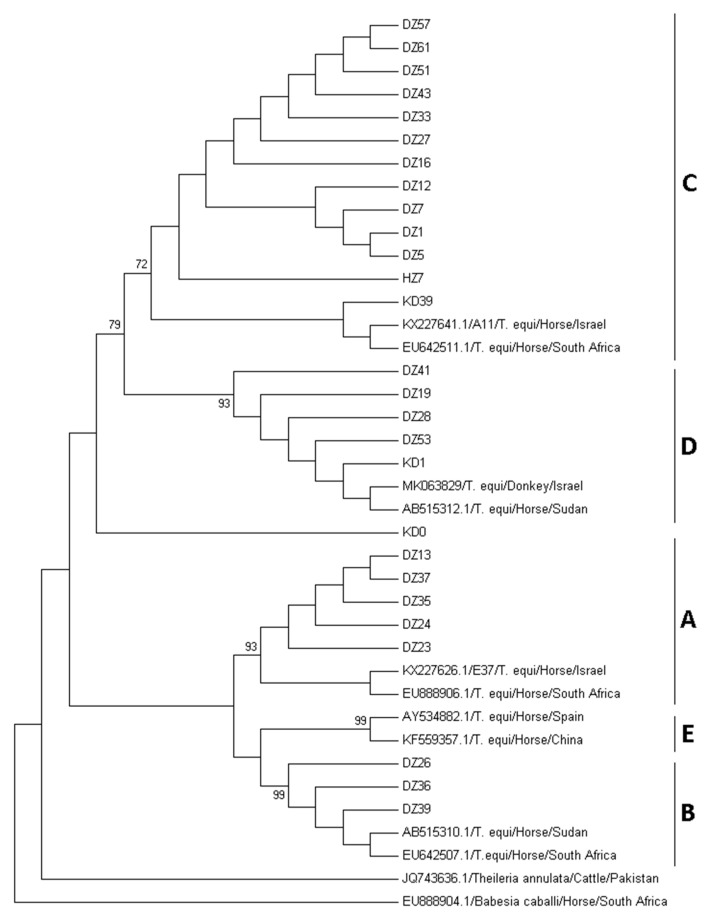
Phylogenetic analysis of *T. equi 18S rRNA* gene from donkeys and horses from Nigeria. The sequences obtained in this study were compared with previously published sequences of *T. equi* and other apicomplexan parasites obtained from the GenBank. The phylogenetic tree, which included 363 positions, was constructed using neighbor joining algorithm and Kimura 2-parameter + G + I model in MEGA7 software. Three samples with shorter sequences were not included in this analysis. Z—Zaria, K—Kaduna, D—Donkey, H—Horse. A–E—the five known *T. equi 18S rRNA* genotypes.

Sequence analysis of the *18S rRNA* gene of PCR positive amplicons from the 25 *T. equi*-infected donkeys (MN093895-MN093919) revealed four different monophyletic clades, comprising seven parasites in clade A, three in clade B, 11 in clade C and four in clade D (Figure 1). Only two of the positive samples were PCR positive for the presence of *ema-1* gene with a 319 bp segment with over 99% homology to *T. equi ema-1* genotype A1 in the GenBank (KX533891.1) isolated from horses in Israel. Ten of the positive samples were also PCR positive for the presence of *T. equi ema-2* gene. Four good-quality 793 bp, sequences (MN519202-MN519205) were obtained from three samples characterized as *18S rRNA* genotype A, and one as genotype B. The four samples were over 99.7% homologous to each other and over 99.5% homologous to *T. equi ema-2* isolates from India (MG874704.1). All five *B. caballi* isolates (MN105077-MN105081) were characterized as *rap-1* genotype A1 (KF059879.1) (Table S1).

### 3.3. Equine Piroplasmosis Infection in Horses

Three horses from Kaduna (7.9%) and one horse from Zaria (14.3%) were infected by *T. equi*, and none by *B. caballi*. Sequence analysis of the PCR positive amplicons (MN093920-MN093923) characterized one parasite as clade A, two as clade C and one as clade D (Figure 1, Table S1).

## 4. Discussion

The high genetic diversity of *T. equi* parasites found in this study highlights the advantages of the use of molecular tools in determining the epidemiology of EP in Nigeria. Since Nigeria acts as a major crossroads of animal transport in West Africa, and Nigeria and most neighboring countries are considered endemic for EP, the genetic characterization of circulating parasites in subclinical animals may shed light on the possible routes of parasite transmission in the area. Nigeria is also a habitat of several of the documented vectors species of EP [6,7], including *Rhipicephalus evetsi* which was the predominant species found on horses in Zaria city (Idoko Sunday Idoko, University of Abuja, unpublished data). Four of the five previously described *T. equi 18S rRNA* genotypes A, B, C and D were found in Nigeria. Few reports describing *T. equi* infection in Africa include genotyping. Reports from the region describe *T. equi 18S rRNA* genotypes A, C and D in neighboring Chad [27], as well as in Tunisia [28]; genotypes A and D in Congo and Senegal [26]; and genotypes B and D in Sudan [29]. Although recent reports suggest that parasite identification and characterization using the *18S rRNA* gene may lead to misclassification of other *Theileria*-like parasites such as *T. equi* [27,30], the fact that all four genotypes that are known to be prevalent in Africa were found in Nigeria supports the hypothesis that animal transport through Nigeria may contribute to the spread of parasites to and from other countries in the region.

Although, the characterization of *T. equi* according to its *18S rRNA* gene has been scrutinized, [27,30], few other genes are routinely used for identification and genotyping of *T. equi*. Most of the focus has been on the immunogenic *ema* gene family [31]. *Ema-1* has been shown to be relatively conserved [32,33], and it is used in the cELISA kit for detection of exposure [34]. However, as in this study, this locus is less sensitive when used in conventional PCR and does not allow discrimination between different strains. *Ema-2,* which has been gaining interest, has also been used for development of an ELISA assay [35]. However, little data exists regarding its polymorphism in different strains. In this study, *ema-2* was successfully sequenced from four of the samples, and although these samples were classified as two distinct *18S rRNA* genotypes (A and B), no heterogeneity was found between different *18S rRNA* genotypes.

*Babesia caballi* parasites were detected in five of the donkeys and were all classified as *rap-1* genotype A1. This genotype has been reported in South Africa [22] and the Middle East [18,19] and also differed in their amino acid sequence from the American genotype, reducing sensitivity for cELISA assays based on this protein in detecting this *B. caballi* strain.

Twenty-nine donkeys were serologically positive to *B. caballi* (29/52) compared to only five that were PCR positive, while similar numbers of donkeys were positive for *T. equi* using both methods (22/52 and 25/57 using IFAT and PCR respectively). *Theileria equi* carriage is considered life long without treatment, while *B. caballi* infection may be cleared months to years following infection [3]. Therefore, in *B. caballi* infection, antibodies may persist after the resolution of parasitaemia, suggesting previous exposure. The use of crude antigen and IFAT method was directed to overcome the detection limitation of the commercial *rap-1* cELISA described in other African and Middle-Eastern countries [18,19,21]. Discrepancies between *T. equi* detection in different methods may be attributed to difference in the sensitivity or efficacy between the methods.

Donkeys in West Africa are mostly used as draught animals and thus are usually kept in more extensive management practices than horses, with no proper surveillance or veterinary care. Moreover, donkeys are considered to be more resistant to clinical disease than horses [36] and may act as asymptomatic reservoirs of EP parasites. Parasite carriage may reduce working capacity of infected donkeys, and carrier donkeys may pose an infection risk for ticks and horses and may spread parasites while transported between countries in animal markets. The donkeys in this study were used as draught animals and although all were sampled in one location, since there was no breeding on the farm, they may have been bought in this type of market and therefore represent a wider range of animals in the area. The relatively high prevalence and variety of parasite strains may support this assumption.

Difference in the prevalence of EP between donkeys and horses in this study is probably due to difference in management and should be evaluated cautiously because of the low number of animals in our sample. Most of the horses in this study were sport horses that were kept in stalls and were well groomed, making them less likely to be exposed to ticks. A larger survey with a more comprehensive sampling design may more reliably estimate EP prevalence in the area.

## 5. Conclusions

To the authors’ knowledge, this is the first molecular study of EP in Nigeria and the first report of EP in donkeys in Nigeria. Our findings demonstrate obvious carriage and exposure rates of donkeys to *T. equi* and *B. caballi*, considerable sequence diversity of *T. equi* isolates, and the presence of a *B. caballi* strain that may not be detected by some cELISA kits. The results of this study suggest that draught donkeys may play a role as reservoir and in the transmission of EP parasites in West Africa.

## Figures and Tables

**Table 1:** The polymerase chain reaction (PCR) primers used in this study.

Primer	Sequence 5′-3′	Target Gene	Amplicon Size (bp)	Reference
Bec-UF2	TCGAAGACGATCAGATACCGTCG	*T. equi/B. caballi* 18S rRNA	400	[21]
Equi-R	TGCCTTAAACTTCCTTGCGAT			
Bc9_RAP2F	ACTAGCGACCCCAACGCTACTGAC	*B. caballi rap-1*	400	[22]
Bc9_RAP2R	TTGGAGCATGAAGTCCTTCAGC			
EMA-1F	GCATCCATTGCCATTTCGAG	*T. equi ema-1*	750	[21]
EMA-1R	TGCGCCATAGACGGAGAAGC			
EMA-2F	AATGTTGAGCAAGTCCTTCG	*T. equi ema-2*	800	[25]
EMA-2R	TTAGTAGAACAAAGCAACGGC			

## Supplementary Materials

The following are available online at https://www.mdpi.com/2076-2615/10/2/324/s1, Table S1: Details of the animals used in this study and identification of *T. equi* and *B. caballi* infection by PCR, genotyping and IFAT.

## References

[1] Hunfeld K.P., Hildebrandt A., Gray J. (2008). Babesiosis: Recent insights into an ancient disease. Int. J. Parasitol..

[2] Onyiche T.E., Suganuma K., Igarashi I., Yokoyama N., Xuan X., Thekisoe O. (2019). A Review on Equine Piroplasmosis: Epidemiology, Vector Ecology, Risk Factors, Host Immunity, Diagnosis and Control. Int. J. Environ. Res. Public Health.

[3] Rothschild C.M. (2013). Equine piroplasmosis. J. Equine Vet. Sci..

[4] Wise L.N., Kappmeyer L.S., Mealey R.H., Knowles D.P. (2013). Review of equine piroplasmosis. J. Vet. Intern. Med..

[5] Margalit Levi M., Tirosh-Levy S., Dahan R., Berlin D., Steinman A., Edery N., Savitski I., Lebovich B., Knowles D., Suarez C.E. (2018). First Detection of Diffuse and Cerebral *Theileria equi* Infection in Neonatal Filly. J. Equine Vet. Sci..

[6] Scoles G.A., Ueti M.W. (2015). Vector ecology of equine piroplasmosis. Annu. Rev. Entomol..

[7] Oguntomole O., Nwaeze U., Eremeeva M.E. (2018). Tick-, flea-, and louse-borne diseases of public health and veterinary significance in Nigeria. Trop. Med. Infect. Dis..

[8] Biu A., Ahmed M., Yunusa A. (2006). Prevalence of equine babesiosis in Maiduguri, Nigeria. Int. J. Biomed. Hlth. Sci..

[9] Ehizibolo D.O., Kamani J., Ehizibolo P.O., Egwu K.O., Dogo G.I., Salami-Shinaba J.O. (2012). Prevalence and significance of parasites of horses in some states of northern Nigeria. J. Equine Sci..

[10] Garba U., Sackey A., Tekdek L., Agbede R., Bisalla M. (2011). Clinical manifestations and prevalence of piroplasmosis in Nigerian royal horses. J. Vet. Adv..

[11] Mshelia W., Sambo K., Adamu S., Edeh E., Onoja I. (2016). Persistence of equine piroplasmosis in horses in Nigeria. J. Equine Vet. Sci..

[12] Sanusi M., Ahmed I.A., Tahir I., Mai H.M., Kalla D.J.U., Shuaibu I. (2014). Survey of equine piroplasmosis in the Savanna areas, Bauchi state, North-eastern Nigeria. Ippologia. Anno.

[13] Turaki U., Kumsha H., Biu A., Bokko P. (2014). Prevalence of Piroplasmosis amongst local horses in Northeastern Nigeria. IOSR J. Agri. Vet. Sci..

[14] Nkala O. (2019). The donkey slaughter of West Africa. Oxpeckers Investigative Environmental Journalism. https://oxpeckers.org/2019/05/donkey-slaughter-capital-of-west-africa/.

[15] Alhassan A., Govind Y., Tam N.T., Thekisoe O.M., Yokoyama N., Inoue N., Igarashi I. (2007). Comparative evaluation of the sensitivity of LAMP, PCR and in vitro culture methods for the diagnosis of equine piroplasmosis. Parasitol. Res..

[16] Munkhjargal T., Sivakumar T., Battsetseg B., Nyamjargal T., Aboulaila M., Purevtseren B., Bayarsaikhan D., Byambaa B., Terkawi M.A., Yokoyama N. (2013). Prevalence and genetic diversity of equine piroplasms in Tov province, Mongolia. Infect. Genet. Evol..

[17] Bhoora R., Franssen L., Oosthuizen M.C., Guthrie A.J., Zweygarth E., Penzhorn B.L., Jongejan F., Collins N.E. (2009). Sequence heterogeneity in the *18S rRNA* gene within *Theileria equi* and *Babesia caballi* from horses in South Africa. Vet. Parasitol..

[18] Mahmoud M.S., El-Ezz N.T., Abdel-Shafy S., Nassar S.A., El Namaky A.H., Khalil W.K., Knowles D., Kappmeyer L., Silva M.G., Suarez C.E. (2016). Assessment of *Theileria equi* and *Babesia caballi* infections in equine populations in Egypt by molecular, serological and hematological approaches. Parasit. Vectors.

[19] Rapoport A., Aharonson-Raz K., Berlin D., Tal S., Gottlieb Y., Klement E., Steinman A. (2014). Molecular characterization of the *Babesia caballi rap-1* gene and epidemiological survey in horses in Israel. Infect. Genet. Evol..

[20] Kappmeyer L.S., Perryman L.E., Hines S.A., Baszler T.V., Katz J.B., Hennager S.G., Knowles D.P. (1999). Detection of equine antibodies to *Babesia caballi* by recombinant *B. caballi* rhoptry-associated protein 1 in a competitive-inhibition enzyme-linked immunosorbent assay. J. Clin. Microbiol..

[21] Alhassan A., Pumidonming W., Okamura M., Hirata H., Battsetseg B., Fujisaki K., Yokoyama N., Igarashi I. (2005). Development of a single-round and multiplex PCR method for the simultaneous detection of *Babesia caballi* and *Babesia equi* in horse blood. Vet. Parasitol..

[22] Bhoora R., Quan M., Zweygarth E., Guthrie A.J., Prinsloo S.A., Collins N.E. (2010). Sequence heterogeneity in the gene encoding the rhoptry-associated protein-1 (RAP-1) of *Babesia caballi* isolates from South Africa. Vet. Parasitol..

[23] Ketter-Ratzon D., Tirosh-Levy S., Nachum-Biala Y., Saar T., Qura’n L., Zivotofsky D., Abdeen Z., Baneth G., Steinman A. (2017). Characterization of *Theileria equi* genotypes in horses in Israel, the Palestinian Authority and Jordan. Ticks Tick Borne Dis..

[24] Steinman A., Zimmerman T., Klement E., Lensky I.M., Berlin D., Gottlieb Y., Baneth G. (2012). Demographic and environmental risk factors for infection by *Theileria equi* in 590 horses in Israel. Vet. Parasitol..

[25] Kumar S., Kumar R., Goyal L., Gupta A.K. (2018). *Theileria equi* equine merozoite antigen-2 (EMA-2) gene (Indian strain) sequence from a Karnal isolate, NCBI GenBank Acc. KC347578.1. https://www.ncbi.nlm.nih.gov/nuccore/KC347578.1.

[26] Edgar R.C. (2004). MUSCLE: Multiple sequence alignment with high accuracy and high throughput. Nucleic Acids Res..

[27] Dahmana H., Amanzougaghene N., Davoust B., Normand T., Carette O., Demoncheaux J.-P., Mulot B., Fabrizy B., Scandola P., Chik M. (2019). Great diversity of Piroplasmida in Equidae in Africa and Europe, including potential new species. Vet. Parasitol. Reg. Stud. Rep..

[28] Ros-Garcia A., M’Ghirbi Y., Hurtado A., Bouattour A. (2013). Prevalence and genetic diversity of piroplasm species in horses and ticks from Tunisia. Infect. Genet. Evol..

[29] Salim B., Bakheit M.A., Kamau J., Nakamura I., Sugimoto C. (2010). Nucleotide sequence heterogeneity in the small subunit ribosomal RNA gene within *Theileria equi* from horses in Sudan. Parasitol. Res..

[30] Knowles D.P., Kappmeyer L.S., Haney D., Herndon D.R., Fry L.M., Munro J.B., Sears K., Ueti M.W., Wise L.N., Silva M. (2018). Discovery of a novel species, *Theileria haneyi* n. sp., infective to equids, highlights exceptional genomic diversity within the genus Theileria: Implications for apicomplexan parasite surveillance. Int. J. Parasitol..

[31] Wise L.N., Kappmeyer L.S., Knowles D.P., White S.N. (2019). Evolution and diversity of the EMA families of the divergent equid parasites, *Theileria equi* and *T. haneyi*. Infect. Genet. Evol..

[32] Bhoora R., Quan M., Matjila P.T., Zweygarth E., Guthrie A.J., Collins N.E. (2010). Sequence heterogeneity in the equi merozoite antigen gene (*ema-1*) of *Theileria equi* and development of an ema-1-specific TaqMan MGB assay for the detection of *T. equi*. Vet. Parasitol..

[33] Manna G., Cersini A., Nardini R., Bartolome Del Pino L.E., Antognetti V., Zini M., Conti R., Lorenzetti R., Veneziano V., Autorino G.L. (2018). Genetic diversity of *Theileria equi* and *Babesia caballi* infecting horses of Central-Southern Italy and preliminary results of its correlation with clinical and serological status. Ticks Tick Borne Dis..

[34] Knowles D.P., Kappmeyer L.S., Stiller D., Hennager S.G., Perryman L.E. (1992). Antibody to a recombinant merozoite protein epitope identifies horses infected with *Babesia equi*. J. Clin. Microbiol..

[35] Kumar S., Kumar R., Gupta A.K., Yadav S.C., Goyal S.K., Khurana S.K., Singh R.K. (2013). Development of EMA-2 recombinant antigen-based enzyme-linked immunosorbent assay for seroprevalence studies of *Theileria equi* infection in Indian equine population. Vet. Parasitol..

[36] Kumar S., Kumar R., Sugimoto C. (2009). A perspective on *Theileria equi* infections in donkeys. Japan. J. Vet. Res..

